# Bio-Innovative Modification of Poly(Ethylene Terephthalate) Fabric Using Enzymes and Chitosan

**DOI:** 10.3390/polym16172532

**Published:** 2024-09-07

**Authors:** Ivana Čorak, Anita Tarbuk, Sandra Flinčec Grgac, Tihana Dekanić

**Affiliations:** Department of Textile Chemistry and Ecology, University of Zagreb Faculty of Textile Technology, Prilaz baruna Filipovića 28a, HR-10000 Zagreb, Croatia; ivana.corak@ttf.unizg.hr (I.Č.); sflincec@ttf.unizg.hr (S.F.G.); tihana.dekanic@ttf.unizg.hr (T.D.)

**Keywords:** PET (poly(ethylene terephthalate)), alkaline hydrolysis, enzymatic hydrolysis, Amano Lipase, chitosan, zeta potential, FTIR, SEM, antimicrobial activity

## Abstract

This article investigates the activation of surface groups of poly(ethylene terephthalate) (PET) fibers in woven fabric by hydrolysis and their functionalization with chitosan. Two types of hydrolysis were performed—alkaline and enzymatic. The alkaline hydrolysis was performed in a more sustainable process at reduced temperature and time (80 °C, 10 min) with the addition of the cationic surfactant hexadecyltrimethylammonium chloride as an accelerator. The enzymatic hydrolysis was performed using Amano Lipase A from *Aspergillus niger* (2 g/L enzyme, 60 °C, 60 min, pH 9). The surface of the PET fabric was functionalized with the homogenized gel of biopolymer chitosan using a pad–dry–cure process. The durability of functionalization was tested after the first and tenth washing cycle of a modified industrial washing process according to ISO 15797:2017, in which the temperature was lowered from 75 °C to 50 °C, and ε-(phthalimido) peroxyhexanoic acid (PAP) was used as an environmentally friendly agent for chemical bleaching and disinfection. The influence of the above treatments was analyzed by weight loss, tensile properties, horizontal wicking, the FTIR-ATR technique, zeta potential measurement and SEM micrographs. The results indicate better hydrophilicity and effectiveness of both types of hydrolysis, but enzymatic hydrolysis is more environmentally friendly and favorable. In addition, alkaline hydrolysis led to a 20% reduction in tensile properties, while the action of the enzyme resulted in a change of only 2%. The presence of chitosan on polyester fibers after repeated washing was confirmed on both fabrics by zeta potential and SEM micrographs. However, functionalization with chitosan on the enzymatically bioactivated surface showed better durability after 10 washing cycles than the alkaline-hydrolyzed one. The antibacterial activity of such a bio-innovative modified PET fabric is kept after the first and tenth washing cycles. In addition, applied processes can be easily introduced to any textile factory.

## 1. Introduction

Polyester accounts for almost 80% of the total production of synthetic fibers, with poly(ethylene terephthalate) (PET) being the most commonly used. Polyester fibers are extremely strong and resistant, but due to their high crystallinity, they are also hydrophobic, which leads to low adsorption capacity (regaining only 0.4%), accumulation of static electricity, soiling and peeling tendency and discomfort when worn. For this reason, surface modifications are carried out to increase the number of hydrophilic groups, usually by hydrolysis with alkalis [1,2,3,4,5,6,7,8] and bio-innovatively with enzymes [9,10,11,12,13,14,15,16,17], aminolysis with amines and newly with ionic liquids [18] or by plasma [17], coating, grafting and UV laser treatment [19].

Hydrolysis causes a cleavage of the ester bonds and leads to an increased number of hydroxyl and carboxyl end groups, so that hydrolyzed fabrics have better adsorption and dyeing properties. Alkaline hydrolysis of the surface of polyester fibers is one of the most commonly used methods in the industry due to its low cost. The process is usually carried out with 4–20% KOH and NaOH at temperatures above 100 °C (130–140 °C) for more than 1 h. It results in a silk-like handle, but also in surface peeling and pitting, which has a negative effect on the strength of the fabric, so the process should be well monitored. In addition, a lot of energy and water are consumed. Some cationic surfactants and polymers (but not all) accelerate the process of alkaline hydrolysis. Originally, the commercially available surfactant Lyogen BPN (fatty acid amine amide from Sandoz) gave the best results [3], but nowadays, quaternary ammonium compounds with at least 16 C atoms, such as cetylpyridinium chloride (CPC), cetyltrimethylammonium bromide (CTAB), hexadecyltrimethylammonium chloride (HDTMAC) and benzalkonium chloride, are used as accelerators [5,6,7,8]. It should be noted that in 1990, Kallay and Grancarić developed a theoretical model for the kinetics of PET decomposition as a function of temperature and the addition of accelerators [3]. The optimum weight loss is between 10 and 24% with a decrease in breaking force of up to 35%, which is achieved by monitoring alkali concentration, time and temperature. Under harsher processing conditions, the pits become cracks and holes, indicating complete damage to the fabric, which then no longer has any useful properties. On the other hand, the monomers required for recycling can be obtained by complete hydrolysis of polyester [1,2,3,4,5,6,7,8,19].

As the process of alkaline hydrolysis is neither ecologically nor energetically friendly, enzymes have been researched in recent years as an alternative to chemical treatment with alkalis. Enzymes are natural and completely biodegradable protein structures and enable the selective processing of polymeric materials at low temperatures. They are biological catalysts that catalyze the polyester hydrolysis reaction under mild conditions and only functionalize the surface. In contrast to alkaline hydrolysis, the enzymes act on the fiber surface due to their size without forming pits or damaging the fiber. Various enzymes are used for the enzymatic hydrolysis of PET: lipase, esterase, cutinase, protease, papain, laccase and glycosidase, with the best effects being achieved with lipase and cutinase. However, their use has been researched mainly for films and foils and only to a limited extent for textiles [9,10,11,12,13,14,15,16,17]. In addition, textiles are heterogeneous and porous, so that wetting and wicking are very different from homogenous films and foils. Under optimized conditions, lipase enzymes from various sources can improve the hydrophilicity and dyeing properties of processed PET fibers while exhibiting good mechanical properties. Among the lipases, Amano Lipase, which can hydrolyze the polyester surface even in a slightly acidic medium, should be highlighted. The effects of enzymes on textiles are very mild and not comparable to the effects of alkaline hydrolysis, so further research is needed [9,10,11,12,13,14,15,16].

**Figure 1:**
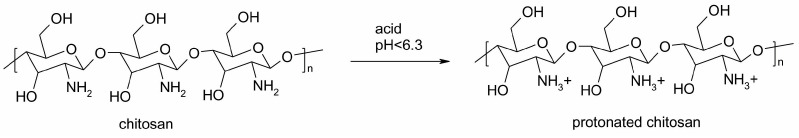
Protonation of chitosan amino groups in acidic medium.

Chitin is the second most abundant polysaccharide in the natural environment after cellulose. Chitin is a structural polymer of the shells of marine invertebrates, the cell walls of fungi and the skeletons of insects and is insoluble in water and most organic solvents. However, enzymatic or chemical deacetylation of chitin produces chitosan, its best-known derivative. Chitosan is a natural biopolymer consisting mainly of 2-amino-2-deoxy-D-glucopyranose units linked by ß-1,4 bonds. It is not soluble in water but is soluble in aqueous acidic solutions. In an acidic medium (Figure 1), the protonation of chitosan amino groups occurs. When alkali is added, it retains the properties of a solution up to a pH value of 6.3, after which it becomes a gel. Studies on the dissolution properties of chitosan and its activation have shown that the dissolution rate varies depending on the acid used. Acetic acid, citric acid, formic acid, 1,2,3,4-butanetetracarboxylic acid, 2,3-dihydroxybutanedioic acid, maleic acid, hydrochloric acid, hyaluronic acid and lactic acid have been used for this purpose [8,20,21,22,23,24,25,26,27,28,29,30,31,32,33,34,35,36,37].

The use of chitosan in textile finishing and fiber spinning is increasing as public awareness and EU policy have highlighted the need for new biological agents to replace existing antimicrobial agents, e.g., quaternary ammonium compounds (QACs), triclosan and others, in the development of hospital materials. Chitosan is characterized by biocompatibility, biodegradability, non-toxicity, antimicrobial, hemostatic and moisturizing properties. Due to its good mechanical and thermoplastic properties, it can be used in medicine as a material for prostheses, blood vessels and artificial organs as well as in systems for the controlled release of drugs and for gene delivery [21,22,23,24,25,26,27,28,29,30,31,32,33,34,35,36,37,38,39,40]. In medicine and healthcare, PET fibers and materials are used in areas where mechanical properties, bio-inertness and durability are critical, such as surgical gowns, membranes, vascular prostheses, stents, reconstructive support meshes, ligaments, tendons and implants. Recently, blends with chitosan at the polymer level have been developed, or PET has been coated with chitosan for medical applications [28,29,30,31,32,33,34,35,36,37,38,39].

The textile application of chitosan depends on the origin of the chitin, the degree of deacetylation, the molecular weight distribution, the concentration and type of acid used, the pH value, the ionic character and the temperature, which all have an important influence on the efficiency of processing textile materials with chitosan [25,26,27]. The antimicrobial efficacy of chitosan is based on the reaction of the NH_3_^+^ groups present with the negatively charged cell membrane of the microbes, and the efficacy of chitosan is greater at low pH [20,38,39,40,41,42]. In addition, the particle size influences the dissolution rate—the smaller the particles, the faster they dissolve in the acid [38,39,40,41,42].

Chitosan can be applied to textiles in various forms, e.g., as gels, membranes, nanofibers, micro- and nanoparticles [8,21,22,23,24,25,26,27,28,29,30,31,32,33,34,35,36,37,38], but mostly, it is not resistant to textile care processes. To improve bonding chitosan on textiles, various crosslinking agents are often used, which can contribute to the stability of chitosan but also affect the desired properties due to the amino reaction group with a crosslinking tool. 

For all these reasons, this article investigated the alkaline hydrolysis of surface groups of PET fibers in woven fabric at a reduced temperature with the addition of an accelerator (HDTMAC) and hydrolysis using the enzyme lipase for surface activation. In contrast to the homogeneous surfaces of PET films and foils, heterogeneous PET fabrics exhibit a dual porosity—an intra- and an inter-yarn porosity—which leads to different interfacial phenomena than with foils or films made of the same polymer. Since all finishing treatments have to be environmentally friendly while fulfilling the main properties of the textiles and have to be durable for at least 3 to 50 washing cycles depending on the use of the textile material, the functionalization with homogenized chitosan gel prepared from submicron particles in acetic acid and its durability in modified industrial washing processes were investigated.

## 2. Materials and Methods

### 2.1. Materials

A commercial polyester fabric (Belira, Banja Luka, Bosnia and Herzegovina, donation), made of 100% poly(ethylene terephthalate) (PET) in a plain weave with a surface mass of 60 g/m^2^, stabilized with hot air, consisting of a textured multifilament yarn of 50 dtex, 16 f, was used for the research.

Oyster mushroom (*Pleurotus ostreatus*) chitosan (Chibio BioTECH, Qingdao City, China), donated by Tricomed SA (Poland), was used in this research. Chitosan has a degree of deacetylation of 90%, a viscosity of 1000 cPs (mPa·s) and a molecular weight of 150 kDa. The sub-micron particles of chitosan (0.5–1 µm) were produced by milling in a Planetary Micro Mill PULVERISETTE 7 premium line (FRITSCH GmbH—Milling and Sizing, Weimar, Germany) using ceramic balls with a diameter of 20 mm for 48 min at 900 rpm. 

### 2.2. Treatment Procedure

The hydrolysis was carried out by a batchwise method in stainless-steel bowls of the laboratory device Linitest (Original-Hanau, Hanau, Germany) with a liquor ratio of 1:50. Hydrolysis was carried out in 1.5 M NaOH (Gram-mol d.o.o., Zagreb, Croatia) at 80 °C for 10 min with the addition of 2 g/L accelerator, cationic surfactant hexadecyltrimethylammonium chloride (HDTMAC, 25% aqueous solution, Sigma-Aldrich Co.-Merck KGaA, Darmstadt, Germany). The samples were washed with tap water and neutralized with 10% acetic acid (acetic acid 80% p.a., Gram-mol d.o.o., Zagreb, Croatia).

Enzymatic hydrolysis was carried out with 0.2 g/L Amano Lipase A from *Aspergillus niger* (Sigma-Aldrich Co.-Merck KGaA, Darmstadt, Germany) at pH 9 and 60 °C for 60 min.

For the functionalization of modified PET fabrics, the homogenized gel of 3 g/L sub-micron particles of chitosan in 3% acetic acid water solution was used. The fabrics were impregnated (padded) in the homogenized chitosan gel solution with wet pick-up (WP) 100%, dried at 110 °C for 2 min and thermocondensed (cured) at 170 °C for 90 s [43]. 

**Figure 2:**
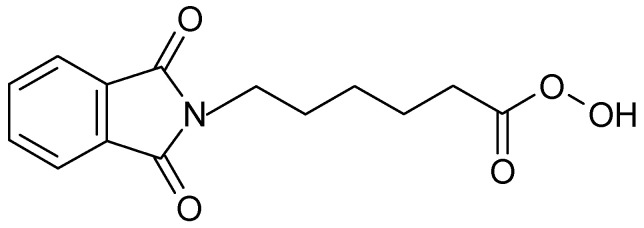
ε-(phthalimido) peroxyhexanoic acid (PAP).

PET fabrics were washed for 1 and 10 cycles in the Wascator FOM71 CLS washing machine (Electrolux, Stockholm, Sweden), applying a modified industrial washing process. According to ISO 15797:2017 *Textiles—Industrial washing and finishing procedures for testing of workwear*, the washing process is carried out at a temperature of 75 °C with peracetic acid [44]. This standard washing process was modified by using soft water with the addition of WFK detergent code 88060 with optical brightener, followed by the addition of ε-(phthalimido) peroxyhexanoic acid (Figure 2), i.e., PAP, (Eureco LX5, Solvay, Brussels, Belgium) as an environmentally friendly agent for chemical bleaching and disinfection. Washing was carried out at a lower temperature of 50 °C, according to a specially developed program. Drying was carried out in a T5130-LAB Type A1 dryer (Electrolux, Stockholm, Sweden) for 30 min in the NORMAL LAB program. 

**Figure 3:**
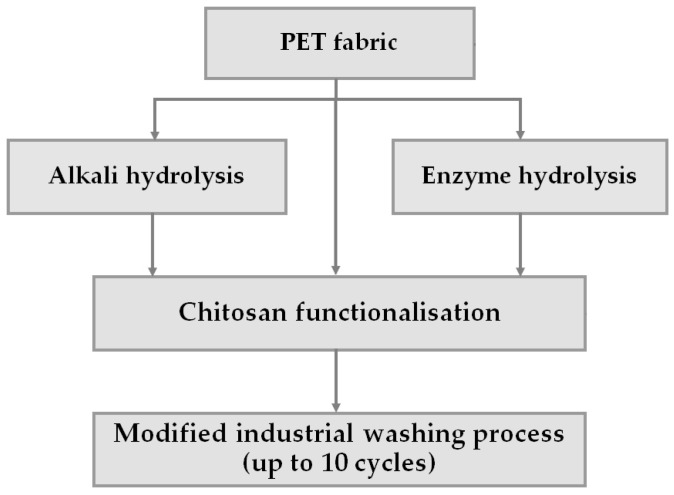
The process line diagram.

The labels and treatments of the PET fabric are listed in Table 1, and the process line diagram is shown in Figure 3.

### 2.3. Characterization Methods

The effectiveness of the implementation of chitosan in polyester fabrics before and after surface activation by hydrolysis and after the 1st and 10th washing cycle was determined using the methods mentioned below.

The mass per unit area (*m*) in g/m^2^ was determined in accordance with ISO 3801:1977 *Textiles—Woven fabrics—Determination of mass per unit length and mass per unit area*, using an analytical balance model ALJ 220-5DNM (KERN & Sohn GmbH, Balingen, Germany) with an accuracy of 0.0001 g. The change in mass per unit area was calculated from the values obtained.

The breaking force (*F*) in N and the elongation (*ε*) as a percentage were determined according to ISO 13934-1:2013 *Textiles—Tensile properties of fabrics—Part 1: Determination of maximum force and elongation at maximum force using the strip method* on a Tensolab dynamometer (Mesdan S.p.A., Puegnago del Garda, Italy) in the warp direction; the distance between the clamps was 100 mm, the width of the sample was 5 cm, the bursting speed was 100 mm/min and the pretension was 2 N. The change in breaking force and elongation was calculated from the values obtained. The tensile index (*TI*), which represents tensile strength (*TS*) over mass per unit area (*m*), was calculated according to (1) using breaking force (*F*) and the width of the sample (*d*) for tensile strength calculation.

(1) $$TI=\frac{TS}{m}=\frac{F/d}{m}\;[Nm/g]$$

AATCC TM 198-2020 *Horizontal Wicking of Textiles* was used to determine the hydrophilicity. Prior to the measurement, the fabrics were conditioned in the constant-climate chamber model KBF-S 240 E6, 9020-0366 (Binder GmbH, Tuttlingen, Germany) at 21 ± 2 °C and a relative humidity of 65 ± 5%.

Samples were analyzed using Fourier transform infrared spectroscopy (FTIR, Perkin Elmer, Spectrum 100 S, Shelton, CT, USA) using the Attenuated Total Reflectance (ATR) technique. Four scans with a resolution of 4 cm^−1^ between 4000 cm^−1^ and 380 cm^−1^ were performed for each sample. When creating the diagrams (Perkin Elmer, Spectrum 100 software, Shelton, CT, USA), the FTIR curves were normalized at 1502 cm^−1^.

The SurPASS electrokinetic analyzer (Anton Paar GmbH, Graz, Austria) was used to determine the electrokinetic potential (*ζ*, zeta, ZP). Based on the measurement of the streaming potential, ZP was calculated according to the Helmholtz–Smoluchowski equation [45,46]. It was measured as a function of the pH value (from pH 9 to pH 2) of 0.001 mol/L potassium chloride (KCl p.a., Kemika, Zagreb, Croatia) using an Adjustable Gap Cell, and the isoelectric point (IEP) was determined. For each point, the streaming potential was measured at 8 points of each sample for each pH value. The pH of the electrolyte solution was adjusted with 0.1 mol/L NaOH (Gram-mol d.o.o., Zagreb, Croatia).

The morphological characterization of the surface of the PET fibers in fabrics was analyzed from micrographs taken with a scanning electron microscope (SEM) (FE-SEM, Mira II, LMU, Tescan, Brno, Czech Republic) with a magnification of 2000× using a combination of two detectors—SE (secondary electron detector) and BSE (backscatter electron detector). The samples were coated with a thin chromium layer for 180 s in a sputter coater Q150T ES Plus (Quorum Technologies, Laughton, UK).

The antimicrobial activity towards Gram-positive bacteria *Staphylococcus aureus* ATCC 6538 (*S. aureus*), Gram-negative bacteria *Escherichia coli* ATCC 8739 (*E. coli*) and microfungi *Candida albicans* ATCC 10231 (*C. albicans*) was determined according to ISO 20645:2004 *Textile fabrics—Determination of antibacterial activity—Agar diffusion plate test*.

## 3. Results and Discussion

The hydrolysis of PET fibers in fabrics was researched to find a more environmentally friendly, economical and/or energetically favorable surface activation process that simultaneously improves hydrophilicity and maintains satisfactory mechanical properties. For this research, hydrolysis was carried out in a more sustainable way: alkaline hydrolysis at a lower temperature and for a shorter time with the addition of an accelerator HDTMAC and bio-innovative hydrolysis using the enzyme lipase were performed. The functionalization of the PET surface with the biopolymer chitosan with minimal damage to the modified PET fabric was also researched.

**Figure 4:**
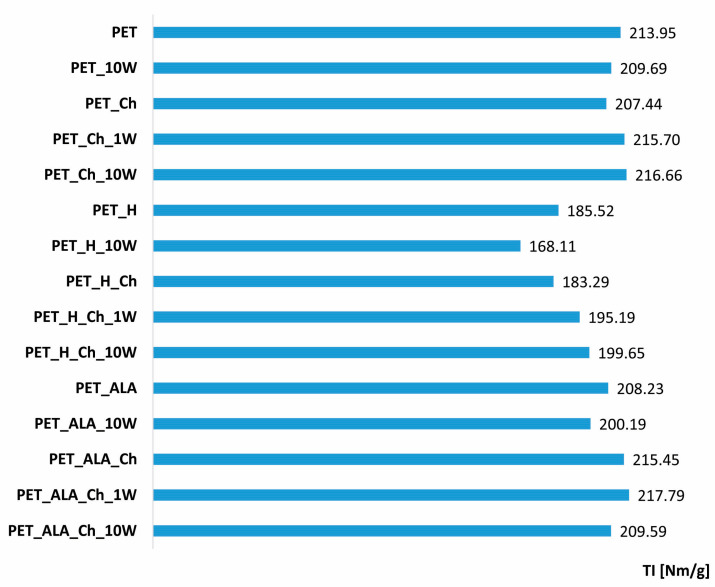
Tensile index (TI) of PET fabrics before and after hydrolysis, chitosan functionalization and 1 and 10 washing cycles.

Table 2 shows the mass per unit area and the tensile properties (breaking force and elongation) of PET fabrics and their changes before and after hydrolysis, chitosan functionalization and one and ten washing cycles. In Figure 4, the tensile index of PET fabrics is presented. It can be seen that the mass of the untreated PET sample is 61.23 g/m^2^. After 10 washing cycles, there is a slight increase in mass (2.18%), probably due to wear and shrinkage of the PET fabric. Alkaline hydrolysis results in a weight loss of 7.04%, caused by the action of alkali on the surface, which leads to peeling and pitting on the surface but is within an optimal range. After 10 washing cycles, a similar slight increase in mass (1.18%) due to shrinkage of the alkaline-hydrolyzed PET fabric can be observed.

Enzyme hydrolysis did not result in weight loss regardless of the effect of the enzyme on the fiber surface. Lipases are biological catalysts that catalyze polyester hydrolysis under mild conditions, but due to their larger molecular size, this reaction only takes place [12,13] on the surface without damaging the fiber or causing pitting. Further SEM micrographs confirm this finding. The enzymatic action only causes a peeling of the surface, which is removed from the fiber during the washing cycles, resulting in a weight loss (1.27%) compared to PET_ALA.

The functionalization of untreated and modified PET fabrics with homogenized chitosan gel leads to an increase in mass per unit area of about 4%. This was to be expected, as chitosan binds to active groups of the PET fibers and forms a chitosan layer on the surface of the PET fabric. 

The washing cycles do not lead to a decrease but to an increase in the mass of PET fabrics functionalized with chitosan compared to untreated PET fabric. It can be seen that the first washing cycle leads to weight loss, as the unbound chitosan has been partially removed from the surface compared to the fabrics after functionalization. However, there is a difference in mass after the tenth washing cycle, which indicates a different durability of the chitosan functionalization. For the unhydrolyzed PET fabric functionalized with chitosan, the mass is similar after 10 washing cycles (PET_Ch_10W); it stays the same as after the first washing cycle, whilst the mass of the alkali-hydrolyzed PET fabrics functionalized with chitosan (PET_H_Ch_10W) have an additional weight loss of 3%, and the ones pretreated with an enzyme (PET_ALA_Ch_10W) have an increase of 1%. The reason for this can be attributed to the removal of chitosan by mechanical and chemical activity during the washing process. It can be assumed that the alkaline medium of the detergent enhanced the effect of alkaline hydrolysis, so the weight loss is higher. As for the PET fabrics pretreated with an enzyme, as the enzyme hydrolysis occurs on the surface due to the size of enzyme [12,13], there is a possibility that chitosan is just redeposited during the washing process. The results of the tensile properties show similar behavior.

Untreated PET has a breaking force of 655 N and an elongation of 29.16%. After 10 washing cycles, there were no significant changes in tensile properties, even when the alkaline detergent was used. There was a 2% change in the tensile index. Functionalization with homogenized chitosan gel has no effect on the strength of the fabric, as the change in tensile strength is only 1%. It even increases to 3.6% during the washing cycles, which indicates possible redeposition of chitosan on the surface and a possible shrinkage of the PET fabric. If the mass per unit area is taken into account when calculating the tensile index, the same behavior can be observed.

As expected, alkaline hydrolysis leads to a significant decrease in breaking force by 19.39% and elongation by 8.44%. Since the acceptable loss of breaking force after hydrolysis according to the literature is up to 40% [1,2,3,4,7], it can be observed that the process was well thought out. After 10 washing cycles, a loss of 6% in tensile strength can be observed, which can be attributed to the alkali medium of the detergent. Taking into account the mass per unit area, the tensile index in alkaline hydrolysis indicates a change of 13.4%, and after 10 washing cycles, of 21.4%. By applying chitosan to its surface, the decrease in breaking force is 16.79% compared to the unhydrolyzed PET fabric but increased by 10% if compared to the alkali-hydrolyzed PET fabric. After the first washing cycle, the chitosan was removed, which is reflected in the lower breaking force compared to the PET_H_Ch sample. Considering the tensile index, the change occurs in the first washing cycle, and after tenth, it is 10% higher. This increase in breaking force and tensile index after 10 washing cycles may indicate shrinkage of the fabric or the accumulation of detergent on the surface due to the washing process.

The enzymatic hydrolysis did not change tensile properties significantly. After 10 washing cycles, there was a decrease in breaking force and tensile index of 5% and 2%, respectively. This change may be attributed to the removal of impurities from the fiber surface resulting from the peeling of the fibers by the action of enzymes. The application of chitosan to the surface resulted in an increase in breaking force compared to the unhydrolyzed sample, confirming the presence of chitosan on the surface. The breaking force increases with the first washing cycle, while it slightly decreases after the tenth washing cycle, but it is still higher than the PET_ALA sample, which may indicate the presence of chitosan particles on the surface as well as the deposition of detergent. The results of the tensile index confirm this behavior.

**Figure 5:**
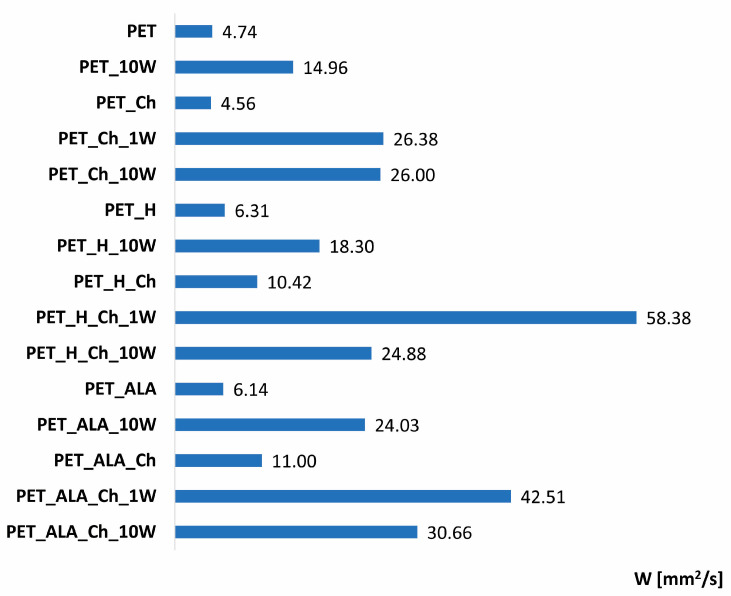
Wicking rate (W) of PET fabrics before and after hydrolysis, chitosan functionalization and 1 and 10 washing cycles.

The most common method for determining the hydrophilic/hydrophobic properties of a solid surface is the drop test with water. When a drop of water settles on the surface, a contact angle is formed. Unlike films and foils, textiles are heterogeneous and porous, so it is sometimes not possible to determine the contact angle, making the wicking test method more suitable [46,47,48]. Since the purpose of PET surface activation is hydrophilicity, the horizontal wicking test was performed according to AATCC TM 198-2020. The results are presented as the wicking rate (W), which represents the calculated water area as a function of time [mm^2^/s] (Figure 5).

Wetting occurs first, followed by wicking, which occurs when the water penetrates the capillary formed by the capillary forces between two fibers or yarns [47,48]. From the wicking rate (W) results shown in Figure 5, it can be seen that PET fabric has a wicking rate of 4.74 mm^2^/s. PET is hydrophobic as a polymer, but in the case of PET fibers in the fabric, some spreading occurs due to the capillary system. Both hydrolyses lead to a higher wetting rate, 6.31 mm^2^/s for alkaline and 6.41 mm^2^/s for lipase hydrolysis, respectively. Chitosan functionalization improves the hydrophilicity, which significantly increases after the first washing cycle. It is possible that redeposition of chitosan occurs and that the amino groups of the chitosan contribute to water spreading. It can also be seen that all washing cycles contributed to a higher wicking rate, so it can be assumed that the water–detergent system leads to better hydrophilicity. After the 10th washing cycle, the results are lower, indicating that some amount of chitosan was washed from the surface.

**Figure 6:**
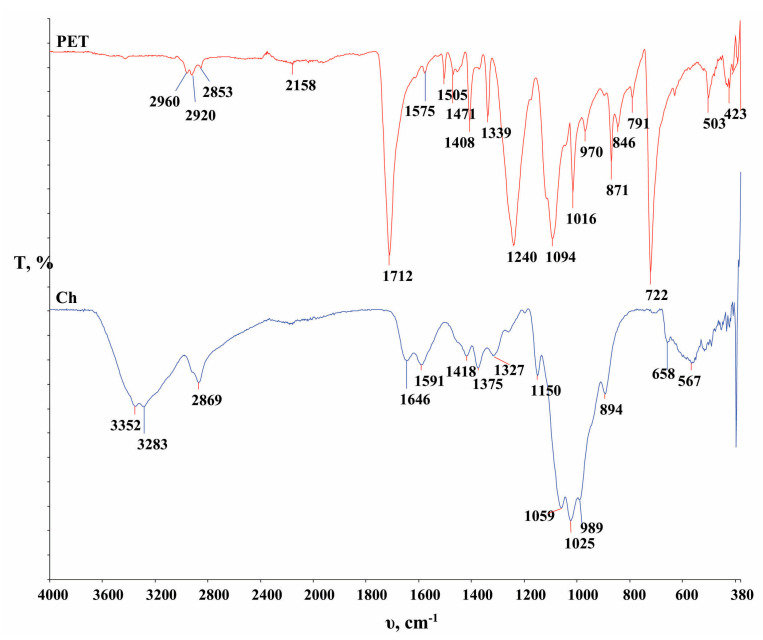
FTIR analysis of untreated polyester fabric (PET) and chitosan powder (Ch) used for this research.

**Figure 7:**
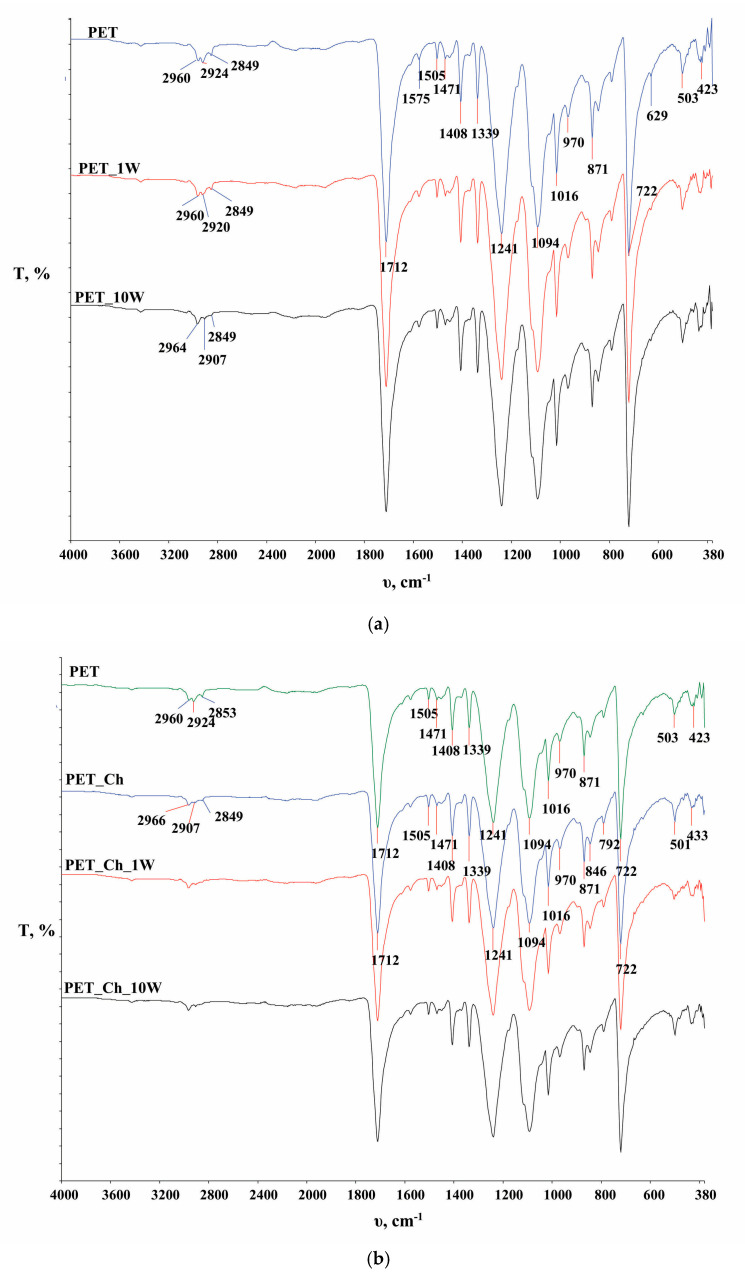
FTIR analysis of untreated polyester fabric (PET) before (**a**) and after chitosan functionalization (PET_Ch) (**b**) and after the 1st (PET_1W, PET_Ch_1W) and 10th (PET_10W, PET_Ch_10W) washing cycle.

**Figure 8:**
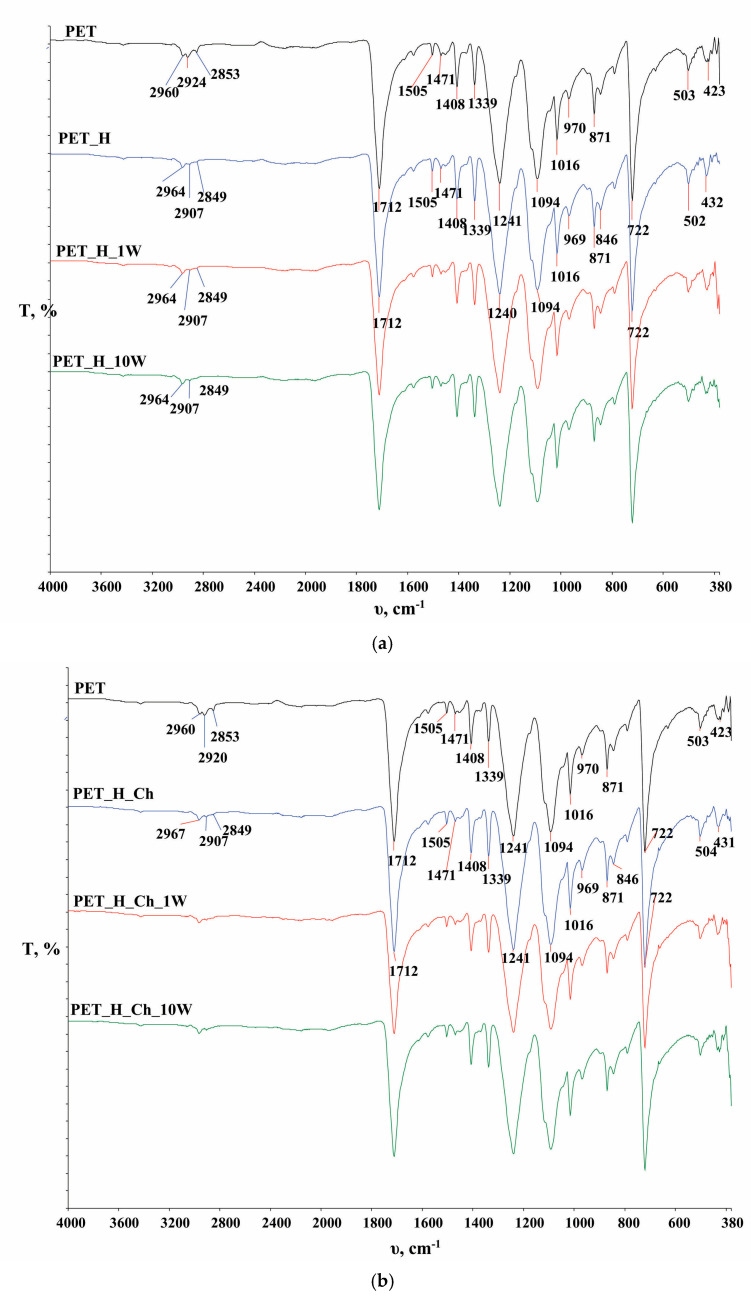
FTIR analysis of alkali-hydrolyzed polyester fabric (PET_H) before (**a**) and after chitosan functionalization (PET_H_Ch) (**b**) and after the 1st (PET_H_1W, PET_H_Ch_1W) and 10th (PET_H_10W, PET_H_Ch_10W) washing cycle.

**Figure 9:**
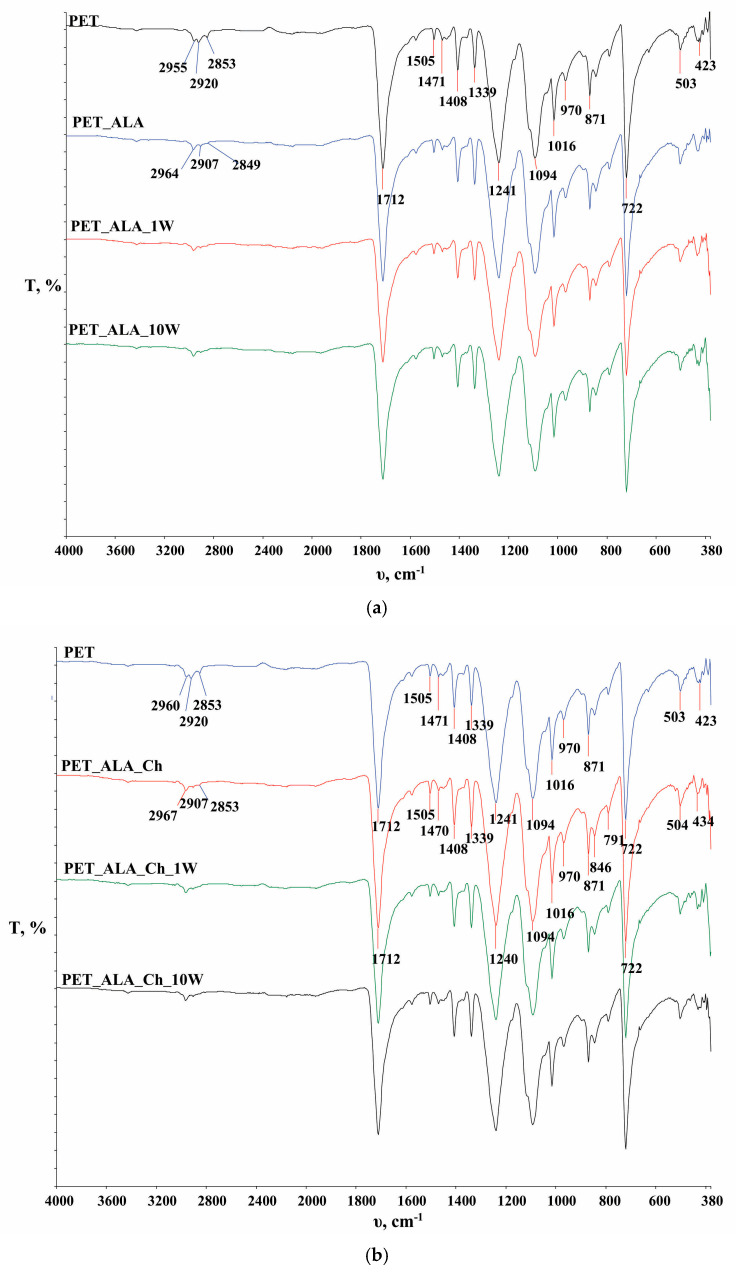
FTIR analysis of enzyme-hydrolyzed polyester fabric (PET_ALA) before (**a**) and after chitosan functionalization (PET_ALA_Ch) (**b**) and after the 1st (PET_ALA_1W, PET_ALA_Ch_1W) and 10th (PET_ALA_10W, PET_ALA_Ch_10W) washing cycle.

In Figure 6, the FTIR spectra of the PET fabric and the chitosan powder are presented, while in Figure 7, Figure 8 and Figure 9, functionalized and washed PET fabrics’ FTIR spectra are shown.

The characteristic bands for PET fabric shown in Figure 6 are as follows. The signal at 2960, 2920 and 2850 cm^−1^ indicates symmetric C–H stretching. The intense signal at 1710 cm^−1^ and 1471 cm^−1^ is due to the stretching of the ester–carbonyl bond (C=O stretching). The signals at 1451 and 1339 cm^−1^ indicate the stretching of the C–O group, the deformation of the O–H group and the bending and wagging modes of the ethylene glycol segment. The signals at 1575, 1505 and 722 cm^−1^ indicate vibrations within the C=C group of the benzene ring. The characteristic signals at 1241, 1094 and 1017 cm^−1^ are the result of C-O, C-O-C and C-OH vibrations, respectively. The peaks at 871 and 846 cm^−1^ are associated with vibrations within the *p*-substituted benzene ring [30,31,49,50,51,52]. In Figure 6, the FTIR spectrum of chitosan powder (Ch) with its characteristic signals is also shown. The intense signal at 3352 and 3283 cm^−1^ is due to O–H stretching vibrations of hydroxyl groups (–OH) superimposed on symmetric and asymmetric stretching vibrations of N–H bonds. 

The signal at 2869 cm^−1^ can be attributed to symmetric and asymmetric C-H stretching. The signals at 1644, 1588 cm^−1^ and 1327 cm^−1^ correspond to the carbonyl group (C=O) (amide I), the bending of the N–H bond (amide II) of the primary amino group and the C-N stretching of amide III in chitosan. A characteristic signal of the CH–OH bond is seen at 1418 cm^−1^, and the CH_2_–OH bond appears at 1375 cm^−1^. The signal at 1150 cm^−1^ corresponds to the asymmetric stretching of the C–O–C bond, and the signals at 1059 and 1025 cm^−1^ are due to the presence of the stretching of the C–O bond [53].

**Figure 10:**
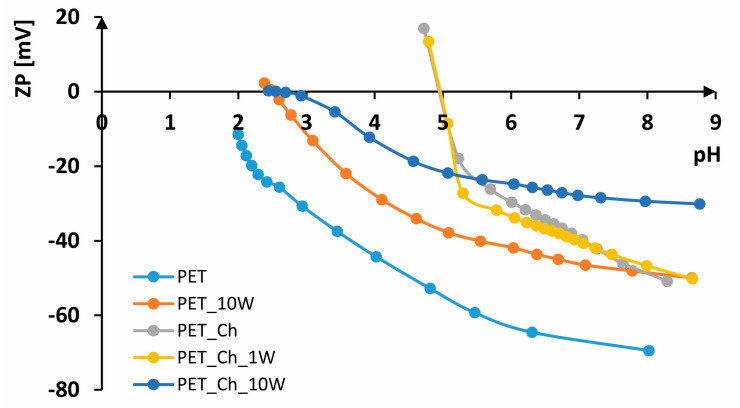
Zeta potential of non-hydrolyzed PET samples.

**Figure 11:**
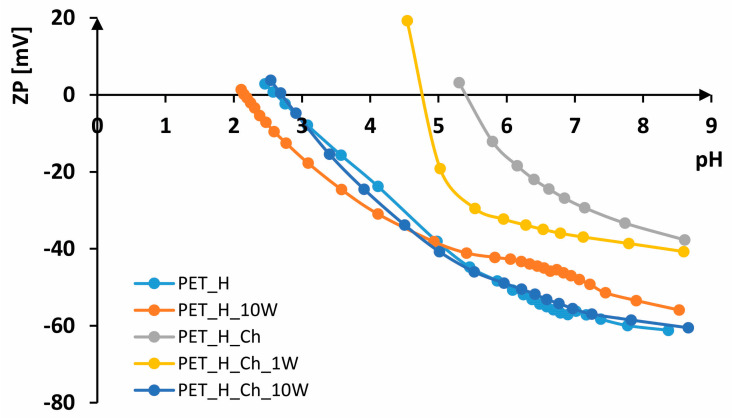
Zeta potential of alkaline-hydrolyzed PET samples.

**Figure 12:**
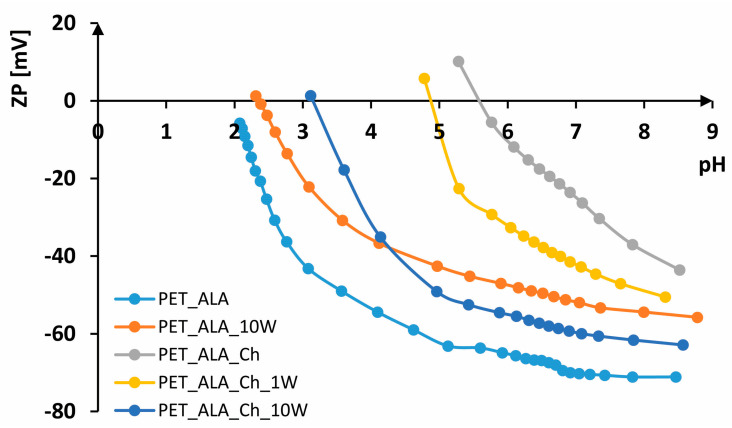
Zeta potential of enzymatically hydrolyzed PET samples.

In Figure 7, Figure 8 and Figure 9, FTIR-ATR spectra of PET fabrics before and after hydrolysis, chitosan functionalization and one and ten washing cycles are shown. In Figure 7, Figure 8 and Figure 9, the changes in the PET fabrics pretreated with NaOH and enzymes and after several washing cycles are presented. The changes are visible by the increase in the peak at 2960 cm^−1^ and the decrease in the peaks at 2920 and 2850 cm^−1^, which are caused by stretching within the CH_2_ groups in the polymer. It is clear from the spectral curves presented for all samples analyzed that the samples treated with chitosan after the pretreatment procedures show no physicochemical changes compared to the pretreated samples, and it can be assumed that the amount of chitosan applied is too small to be detected by this method. Therefore, the samples were characterized by their zeta potential. The results are presented in Figure 10, Figure 11 and Figure 12. 

The electrokinetic analysis was performed by measuring the zeta potential (ζ) on SurPASS. From the results shown in Figure 10, it can be seen that untreated PET fabric has a zeta potential of ζ = −69.46 mV at a pH of 8.02, IEP < 2. The zeta potential of PET fabric in an alkaline medium is due to its hydrophobic surface. Due to the high crystallinity of the fibers, the number of carboxyl groups is very low, which makes the surface of the fiber hydrophobic. In contrast to hydrophilic surfaces, which have a higher zeta potential due to their ability to absorb water, hydrophobic surfaces have a lower zeta potential since they cannot absorb water molecules [8,46].

According to the literature, their zeta potential varies from ζ = −40 mV to ζ = −80 mV depending on the structural parameters [8,54]. During washing [45,55], the fabric is worn down, resulting in fiber damage and fibrillation, so that more negative groups are available. However, considerable shrinkage of the fabric leads to a higher zeta potential in alkaline and neutral media, which is why the zeta potential is not more negative after several washing cycles. According to that, after 10 washing cycles, the PET fabric exhibits ζ = −49.96 mV at pH 8.65, ζ = 2.28 at pH 2.38 and IEP = 2.5. The use of chitosan leads to a higher zeta potential in alkaline and neutral electrolyte solutions and to a shift of the isoelectric point to a higher pH value. The higher zeta potential is due to the presence of amino groups in chitosan [30,56]. The PET_Ch sample exhibits ζ = −50.82 mV at pH 8.29, which is more positive than unhydrolyzed PET fabric (difference of 20 mV). A zeta potential of ζ = 26.65 mV at pH 4.12 and an IEP of 4.97 indicate the presence of chitosan on the surface. After the first washing cycle, there is no significant change in the zeta potential; at pH 8.66, ζ = −50.18 mV; at pH 4.79, ζ = 25.21 mV; and IEP = 4.96, indicating the stability of the treatment. After the tenth washing cycle, the fabric remains more positive, ζ = −30.12 mV at pH 8.23. However, the removal of chitosan from the surface of the fabric occurred, and the IEP was 2.59, which is almost the same as the PET_10W sample.

Alkaline-hydrolyzed PET fabric at pH 8.37 has ζ = −61.15 mV and IEP = 2.62. The hydrolysis of polyester increases the number of surface-active groups, i.e., carboxyl groups, which leads to a more negative zeta potential in the alkaline electrolyte solution compared to non-hydrolyzed PET [56,57]. The PET_H_10W sample has ζ = −55.88 mV at pH 8.53, while at pH 2.11, it has ζ = 1.35 mV, with an IEP of 2.15. This more positive zeta potential of 5 mV after 10 washing cycles was found in the alkaline electrolyte solution compared to the PET_H sample, probably due to additional pilling during washing, which corresponds to loss of braking force. The PET_H_Ch sample has ζ = −37.67 mV at pH 8.61, while at pH 5.30, it has ζ = 3.22 mV, with an IEP of 5.4. Alkaline hydrolysis of polyester led to the formation of a larger number of active groups to which chitosan was bound, resulting in a more positive zeta potential in the alkaline electrolyte solution and a shift of the IEP to a higher pH value compared to non-hydrolyzed polyester. The first wash led to a partial removal of chitosan, resulting in a leftward shift of the IEP to 4.81. However, the zeta potential at pH 8.59 did not change significantly, with ζ = −40.70 mV, while at pH 4.54, it is ζ = 19.29 mV. The PET_H_Ch_10W sample has almost the same behavior as the PET_H sample; at pH 8.66, it has ζ = −60.49 mV, while at pH 2.54, it has ζ = 3.79 mV, with an IEP of 2.7, which means that after 10 washing cycles, the chitosan has been completely removed from the surface of the hydrolyzed fabric.

Enzymatically hydrolyzed fabric at pH 8.46 has ζ = −71.11 mV, while at pH 2.08, it has ζ = −5.77 mV, with IEP < 2, indicating a larger number of groups on the sample compared to the alkaline-hydrolyzed sample. In an acidic electrolyte solution, the enzymatically hydrolyzed sample is more electronegative than the untreated sample and also than the alkaline-hydrolyzed sample. After 10 washing cycles, the fabric also shrinks, which is reflected in a more positive curve at both alkaline and acidic pH values. For example, the PET_ALA_10W sample has ζ = −55.74 mV at pH 8.78, ζ = 3.21 mV at pH 2.25 and an IEP of 2.35, which is very similar to non-hydrolyzed PET. The PET_ALA_Ch sample has ζ = −43.57 mV at pH 8.52, ζ = 10.13 mV at pH 5.28 and an IEP of 5.59, indicating the presence of chitosan on the surface, as evidenced by both a more positive charge and a shift in the isoelectric points. After the first wash, the chitosan is also partially removed from the surface of the sample; as with the alkaline-hydrolyzed samples, the values at pH 8.31 are ζ = −50.51 mV, while at pH 4.78, they are ζ = 5.78 mV, with an IEP of 4.88. The sample PET_ALA_Ch_10W has ζ = −62.86 mV at pH 8.57, while at pH 2.79, it has ζ = 11.21 mV, with an IEP of 3.15, which indicates the removal of chitosan from the surface of the material after ten washing cycles. The enzymatically hydrolyzed sample exhibits better wash resistance compared to the alkaline-hydrolyzed and non-hydrolyzed sample, as indicated by the shift of its IEP to a higher pH value, suggesting that chitosan is not completely removed from the surface even after 10 washing cycles.

**Figure 13:**
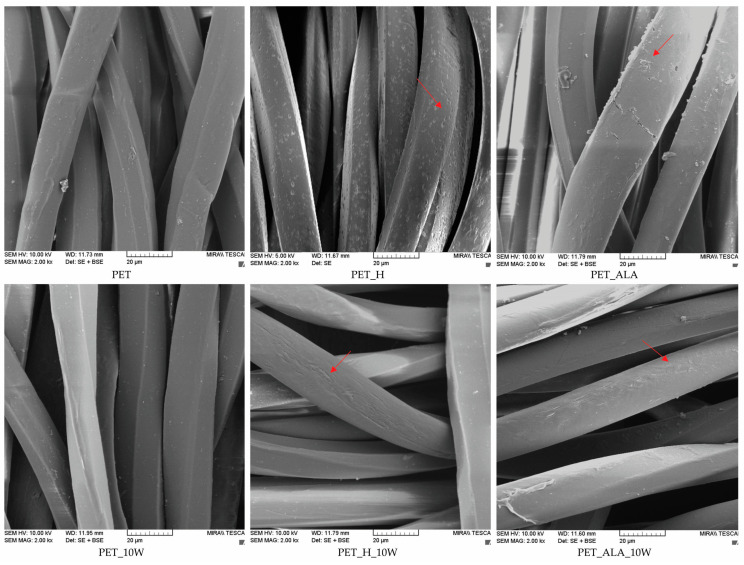
SEM micrographs of unhydrolyzed and hydrolyzed PET fibers in fabrics at a magnification of 2000×, after the modification and 10 washing cycles.

**Figure 14:**
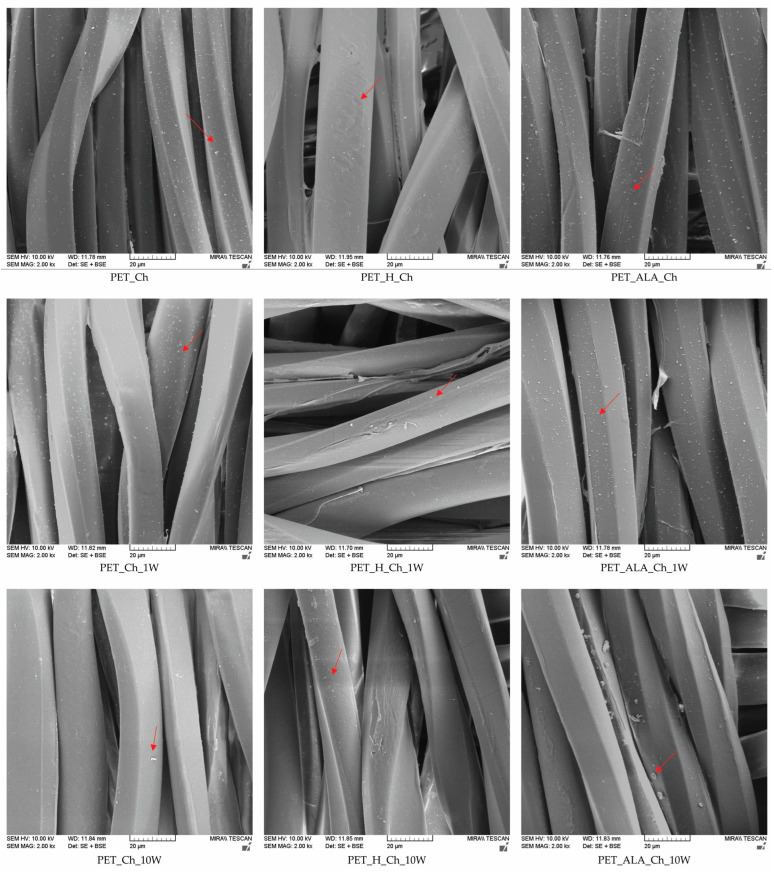
SEM micrographs of chitosan-functionalized PET fabrics at a magnification of 2000×, after the 1st and 10th washing cycle.

In Figure 13 and Figure 14, the micrographs of the PET fibers in fabric taken with a scanning electron microscope (SEM) at a magnification of 2000× are shown, and the morphological characterization of the surface of the PET fibers was performed. It can be seen that the PET sample has a smooth surface with some impurities on the fiber. After 10 washing cycles, the impurities are removed from the fiber surface. The application of chitosan leads to the appearance of granular structures on the surface, indicating the presence of chitosan on the fiber surface, which is confirmed by an increase in surface mass, breaking force and zeta potential. The granular structures on the surface are also visible after the first washing cycle, confirming the stability of the treatment, which was also visible by measuring the zeta potential. The PET_Ch_10W sample shows a clean, smooth structure with no particles on the fiber surface, indicating that the chitosan was removed from the fiber surface as previously confirmed.

The alkaline hydrolysis results in characteristic pits on the surface of the fibers [4,7], which are caused by the action of alkali, as can also be seen from the decrease in breaking strength compared to the non-hydrolyzed sample. The application of chitosan causes it to penetrate into the pores created by the alkali, which is visible on the PET_H_Ch sample. The washing process reduces the number of chitosan particles on the fiber.

The enzymatic hydrolysis led to an exfoliation of the fiber surface, resulting in more free active groups, but the strength was not affected. After 10 washing cycles, the surface of the fibers is cleaned, which correlates to the drop in the breaking force of 5%, although the peeling caused by the enzymatic action is still visible in some places. In the enzymatically hydrolyzed sample, a large number of particles are visible on the fiber surface after the application of chitosan, confirming the presence of chitosan on the fiber that remained after the first washing cycle. The number of particles is visually higher on the enzymatically hydrolyzed sample than on the alkaline-hydrolyzed sample, which correlates with the zeta potential results. After 10 washing cycles, particles are still visible on the fiber surface, which could be chitosan residues or impurities that have accumulated during the washing process.

The antimicrobial activity of PET fabrics before and after hydrolysis, after functionalization with chitosan and after 1 and 10 washing cycles was determined according to EN ISO 20645:2004 against the following microorganisms: Gram-positive bacteria *Staphylococcus aureus* (*S. aureus*), Gram-negative bacteria *Escherichia coli* (*E. coli*) and microfungi *Candida albicans* (*C. albicans*). The results are presented in Table 3.

The evaluation of antimicrobial activity includes the observation of inhibition zones, if present, or the growth of colonies beneath the sample. If the inhibition zone is present or there are no colonies beneath the sample in the contact area, the material has antimicrobial activity. The untreated fabric shows no antimicrobial activity against the tested microorganisms. After 10 washing cycles, antimicrobial activity against *S. aureus* and *E. coli* is present, so that the applied washing process with PAP has contributed to the antibacterial activity as a disinfectant. 

When treated with chitosan, all fabrics showed activity against all microorganisms tested. Better activity can be observed against the Gram-positive bacteria *S. aureus*, and the difference between the fabrics functionalized with chitosan is clearly visible. When chitosan was applied to PET fabric or enzyme-hydrolyzed PET fabric, there was no zone of inhibition. 

However, when PET fabric was activated by accelerated alkali hydrolysis with HDTMAC, an inhibition zone was observed, which persisted even after chitosan functionalization. The reason for this may be in the HTDMAC used for activation, which also acts as an antimicrobial agent. Since the functionalization with chitosan was performed directly after hydrolysis and only one rinse cycle was performed in between to remove the oligomers, it is possible that a certain amount of HDTMAC remained adsorbed on the fabric surface, as suggested by the IEP of PET_H fabric, and provided additional antimicrobial activity. 

The antibacterial activity achieved is maintained after the first and tenth washing cycles, but the effect against microfungi is removed after the first one for PET_Ch and PET_H_Ch fabrics. For enzyme-hydrolyzed fabric (PET_ALA_Ch), antimicrobial activity against microfungi remains after the first washing cycle, indicating a sufficient amount of chitosan in the fabric structure, as suggested by measuring the zeta potential. 

## 4. Conclusions

In this article, the bio-innovative modification of PET fabric using enzymes and chitosan was investigated. Alkaline and enzymatic hydrolysis were carried out to activate the surface of polyester fabric, which was subsequently functionalized with chitosan. The presented results indicate the effectiveness of both hydrolyses. The alkaline hydrolysis led to a loss of strength, while the mechanical damage to the PET fabric was not detected by the action of the enzyme. Enzymatic bioactivation of the surface is also more environmentally friendly as it does not require sodium hydroxide and can be carried out at lower temperatures. The presence of chitosan on the polyester fibers was confirmed by measuring the zeta potential and is visible on the SEM images. In addition, the chitosan functionalization on the bioactivated surface showed better durability after 10 washing cycles, and the antibacterial activity achieved is maintained after the first and tenth washing cycles. In contrast to other environmentally friendly activation processes, such as plasma, this process can easily be introduced in any textile factory.

## Figures and Tables

**Table 1:** Labels and treatment of the PET fabric.

Label	Treatment
PET	Untreated PET fabric
_H	Alkaline hydrolysis with accelerator HDTMAC
_ALA	Enzymatic hydrolysis with Amano Lipases
_Ch	Chitosan functionalization
_1W or _10W	First or tenth washing cycle

**Table 2:** Mass per unit area, breaking force and elongation of PET fabrics and their changes before and after hydrolysis, chitosan functionalization and 1 and 10 washing cycles.

Sample	m [g/m^2^]	Δm [%]	F [N]	ΔF [%]	ε [%]	Δε [%]
PET	61.23	-	655.00	-	29.16	-
PET_10W	62.57	2.19	656.00	0.15	33.55	15.05
PET_Ch	64.02	4.56	664.00	1.37	31.80	9.05
PET_Ch_1W	62.91	2.74	678.50	3.59	31.90	9.40
PET_Ch_10W	62.68	2.37	679.00	3.66	30.60	4.94
PET_H	56.92	−7.04	528.00	−19.39	26.70	−8.44
PET_H_10W	57.64	−5.86	484.50	−26.03	27.80	−4.63
PET_H_Ch	59.47	−2.87	545.00	−16.79	27.42	−5.97
PET_H_Ch_1W	59.02	−3.61	576.00	−12.06	24.26	−16.80
PET_H_Ch_10W	57.25	−6.50	571.50	−12.75	30.00	2.88
PET_ALA	62.72	2.43	653.00	−0.31	35.48	21.67
PET_ALA_10W	61.94	1.16	620.00	−5.34	32.60	−6.65
PET_ALA_Ch	63.54	3.77	684.50	4.50	30.45	4.42
PET_ALA_Ch_1W	64.74	5.73	705.00	7.63	32.00	9.77
PET_ALA_Ch_10W	65.08	6.29	682.00	4.12	32.80	12.48

**Table 3:** The antimicrobial activity of fabrics before and after hydrolysis, functionalization with chitosan, and 1 and 10 washing cycles.

Fabric	*S. aureus*	*E. coli*	*C. albicans*
PET	−	−	−
PET_10W	+/−	+/−	−
PET_Ch	+/−	+/−	+/−
PET_Ch_1W	+/−	+/−	−
PET_Ch_10W	+/−	+/−	−
PET_H	+	+/−	−
PET_H_10W	+/−	+/−	−
PET_H_Ch	+	+/−	+/−
PET_H_Ch_1W	+/−	+/−	−
PET_H_Ch_10W	+/−	+/−	−
PET_ALA	+/−	+/−	−
PET_ALA_10W	+/−	+/−	−
PET_ALA_Ch	+/−	+/−	+/−
PET_ALA_Ch_1W	+/−	+/−	+/−
PET_ALA_Ch_10W	+/−	+/−	−

+ Antimicrobial activity (inhibition zone can be observed); +/− antimicrobial activity (no colonies beneath); − no antimicrobial activity.

## Data Availability

Data available in a publicly accessible repository.
